# Acetabular roof lesions in children: a descriptive study and literature review

**DOI:** 10.1186/s12891-020-03601-x

**Published:** 2020-08-24

**Authors:** Jinkui Wang, Zhongliang Wang, Jiaqiang Qin

**Affiliations:** 1 Department of Orthopedics, Children’s Hospital of Chongqing Medical University, 2 ZhongShan Rd, ChongQing, 400013 China; 2 Ministry of Education Key Laboratory of Child Development and Disorders, Children’s Hospital of Chongqing Medical University, Chongqing, PR China; 3 National Clinical Research Center for Child Health and Disorders (Chongqing), Children’s Hospital of Chongqing Medical University, Chongqing, PR China; 4 China International Science and Technology Cooperation base of Child development and Critical Disorders, Children’s Hospital of Chongqing Medical University, Chongqing, PR China; 5 Chongqing Key Laboratory of Pediatrics, Children’s Hospital of Chongqing Medical University, Chongqing, PR China; 6 Children’s Hospital of Chongqing Medical University, Chongqing, PR China

**Keywords:** Acetabular roof lesion, Children, Eosinophilic granuloma, Bone cyst, Chronic osteomyelitis

## Abstract

**Background:**

Acetabular roof lesions (ARLs) in children are uncommon and may involve a variety of diseases. The acetabular roof is the main weight-bearing area of the hip joint, and lesions affecting the acetabular roof lead to fluid accumulation in the hip joint, causing hip pain and claudication. Methods for diagnosing and treating ARLs and the prognosis after treatment are rarely reported. We present our experience in a group of children and teenagers with ARLs to retrospectively explore the clinical and imaging features and histopathological diagnosis and report the treatment methods and follow-up observations.

**Methods:**

Patients with ARLs admitted to the Children’s Hospital of Chongqing Medical University from April 2011 to September 2018 were selected retrospectively. We collected the basic information of patients (name, sex, age), main symptoms and signs, results of various laboratory tests, treatment methods, and intraoperative observations through the hospital medical record system. We collected X-ray, computed tomography (CT), magnetic resonance imaging (MRI) and pathological examination data through the Picture Archiving and Communications System. Follow-up data were collected through an outpatient medical record system, telephone, and chat software (such as WeChat). We used descriptive methods to analyze the lesion structure and destruction mode based on the imaging findings and histopathological diagnosis.

**Results:**

There were 14 ARL patients, including 6 with eosinophilic granuloma (EG), 5 with chronic osteomyelitis, 2 with bone cyst, and 1 with tuberculosis. One patient underwent percutaneous needle biopsy, 2 underwent open biopsy, and 11 underwent curettage; among them, 5 patients also underwent bone grafting. These lesions had no characteristic imaging findings, and the diagnosis was mainly based on histopathological examination. Most patients showed complete symptom resolution and good hip function at the 1-year follow-up.

**Conclusion:**

ARLs are not common in children. The types of lesions are diverse and mostly benign, with EG being most common. Malignant tumors may also occur, such as Ewing’s sarcoma, non-Hodgkin’s lymphoma, metastases and neuroblastoma. CT and MRI can be helpful in diagnosing certain cases, but incisional biopsy is required in most cases.

## Background

Acetabular roof lesions (ARLs) are rare in children and adults. ARLs mainly involve area II based on the Enneking and Dunham classification [1], and some ARLs can involve area I. The hip joint is the largest weight-bearing joint in the body, and the acetabular roof accounts for the largest part of the acetabular weight-bearing capacity. ARLs can reduce the hardness and load-bearing capacity of the acetabulum. When standing or walking, ARLs might cause acetabular collapse or pathological dislocation under the pressure of the femoral head in severe cases. Acetabular lesions in children may also damage the acetabular triradiate cartilage, cause acetabular dysplasia and affecting acetabular function.

ARLs have rarely been reported in the literature, with only a few case reports of iliac lesions and few reports of cases involving acetabular roof destruction. One article reported 3 cases of eosinophilic granuloma (EG) of the pelvis in children [2]. Two of the patients had lesions located on the acetabular roof. One patient underwent needle biopsy, and one patient underwent incisional biopsy. Hip pain was relieved after biopsy, and no other treatment was performed. The children were completely healed after 1 year. Howard et al. [3] reported three patients with EG of the ilium; the acetabular roof was affected in only 1 case, which was initially misdiagnosed as Ewing’s sarcoma (ES) and was cured after incisional biopsy and curettage of the lesion, with no pain or signs of recurrence. Sun et al. [4] reported 22 cases of iliac bone destruction in children. The lesion types included neuroblastoma (NB) iliac bone metastasis, EG, ES, osteomyelitis, BC, fibrous dysplasia, and non-Hodgkin’s lymphoma (NHL). The definitive diagnosis of these patients was difficult to achieve on imaging, and most of the diagnoses were confirmed by pathological biopsy. Two articles reported acetabular lesions in adults. Prasad et al. [5] reported a 40-year-old female patient with a desmoid tumor in the acetabular roof, and Bauones et al. [6] reported 3 cases of acetabular bone metastasis. However, there have been no articles reporting acetabular roof destruction in children. This article retrospectively explored 14 cases collected from April 2011 to September 2018 at the Children’s Hospital of Chongqing Medical University. The clinical, laboratory and imaging findings and pathological diagnosis in these 14 cases were analyzed retrospectively. We summarized the common types of ARLs and the diagnosis and treatment of various lesions, aiming to improve the diagnosis and treatment of ARLs.

## Methods

Patients were eligible for inclusion if they had a lesion on the acetabular roof between 2011 and 2018. This research was approved by the Ethics Review Board of the Children’s Hospital of Chongqing Medical University. All patients or their relatives provided written informed consent to be included in this study. The inclusion criteria were as follows: (1) age less than 18 years; (2) X-ray images showing lesion involvement of the acetabular roof; (3) exact pathological diagnosis; (4) complete case and imaging data; and (5) more than 1 year of follow-up. The exclusion criteria were as follows: (1) children with acetabular roof fractures caused by trauma; (2) no pathological diagnosis or incomplete information; and (3) recurrence after various prior hip operations. We collected the clinical and imaging data, treatment methods, and follow-up results of the patients.

The main clinical features recorded were age, sex, symptoms (pain, claudication), and physical signs (swelling, tenderness, hip range of motion, local skin temperature and body temperature). We obtained the results of some laboratory examinations performed on admission, including routine blood parameters, the procalcitonin (PCT) and high-sensitive C-reactive protein (CRP) levels, and the erythrocyte sedimentation rate (ESR). Imaging studies of the lesion included X-ray examination in all patients, conventional and 3D computed tomography (CT) in 12 patients, and magnetic resonance imaging (MRI) in 8 patients. The size of the lesion was mainly evaluated on X-ray examination. According to the proportion of the orthotopic lesion in the acetabular roof, they were divided into small (< 1/3), medium (1/3–2/3), large (> 2/3), and extra large (beyond the acetabular roof, up to the iliac wing). Additionally, the status of the margin (with or without sclerotic margination), boundary (clear or unclear), cortical destruction (erodent, thin), periosteal reaction, joint space (widened or narrowed), femoral head size (changed or osteoporotic), and hip joint (dislocated) was considered. Further examination by MRI can allow the evaluation of surrounding soft tissue edema, joint cavity effusion and effects of the lesion on the joint cavity, and CT can be used to evaluate cortical continuity in a more detailed manner.

If the child could not be diagnosed based on clinical, laboratory, and imaging examinations, a needle biopsy or incisional biopsy was performed. Indications for incisional biopsy included the following: (1) no signs of malignancy on preoperative examination; (2) cystic lesions, due to the low success rate of needle biopsy with such lesions; (3) lesions with a maximum diameter > 3 cm (those prone to pathological fracture); (4) the need for bone grafting during surgery; and (5) insufficient tissue provided by needle biopsy for a definitive diagnosis [7]. If the patient had a large lesion and infection and malignant lesions were excluded, artificial bone or allograft bone transplantation was performed to promote healing.

Postoperative assessments were performed at 1, 3, 6 months and 1 year and then annually thereafter. Postoperative follow-up was performed by an independent orthopedic surgeon, who recorded the clinical and imaging data of each patient during follow-up. The imaging assessment included X-ray examination of the lesion; if this clinical assessment was insufficient, CT or MRI was performed. The d’Aubigné-Postel scoring system was used for the clinical assessment of hip function [8] according to the following criteria: pain (scored from 0, permanent pain intensity, to 6, no pain); mobility (from 0, ankylosis with poor hip positioning, to 6, more than 90° of flexion and 30° pf abduction); and ability to walk (from 0, none, to 6, normal).

## Results

### Clinical and imaging features

The study ultimately involved a retrospective review of 14 ARL patients, including 2 females and 12 males, with an average follow-up of 2.1 years (1–8 years). The mean age was 6.3 ± 4.7 years (range, 1–17.3 years). The modified Harris Hip Score (MHHS) of children on admission is shown in Table 1 (less than 90 for all patients). Among these patients, 13 had solitary lesions, and 1 had multiple lesions as preoperatively diagnosed by a skeletal survey. Among these cases, the ARL was on the left side in 6 cases and the right side in 8 cases; in the 1 case of right acetabular EG, there were multiple lesions, including lesions affecting the skull and liver. The disease course ranged from 10 days to 2 years, with an average of 6 months. There were 6 cases of hip pain, 3 cases of claudication, and 5 cases of both hip pain and claudication as initial symptoms. Local pain or tenderness was observed in 6 patients, hip joint limitation was observed in 6 patients. All children had no local increase in skin temperature, no palpable mass, and no fever. Six patients had an elevated white blood cell (WBC) count, 3 patients had an elevated CRP level, 3 patients had an elevated PCT level, and 6 patients had an elevated ESR. A summary of the clinical and laboratory findings for the 14 patients is shown in Table 1.

**Table 1:** Clinical characteristics of the ARL patients (following the Results)

Patient	Sex/age	Lesion type	Clinical features	Treatment	Follow-up (mo)/status	Postoperative d’Aubigné-Postel score
A	F/4.3	EO	Hip pain, swelling, and restricted motion	Lesion curettage, bone allograft	96/healed	17
B	M/10.3	EO	Hip pain and restricted motion	Lesion curettage, bone allograft	24/healed	17
C	M/4.8	EO	Hip pain, claudication, tenderness, and restricted motion	Lesion curettage, bone allograft	12/healed	17
D	M/9.3	EO	Hip pain and tenderness	Lesion curettage, bone allograft	12/healed	17
E	M/2.2	EO	Hip pain and claudication	Lesion curettage, bone allograft	84/healed	17
F	M/1	EO	Hip pain, claudication and tenderness	Open biopsy, chemotherapy	24/healed	18
G	M/3.1	CO	Claudication	Needle biopsy	36/healed	18
H	M/4.7	CO	Hip pain, claudication and restricted motion	Lesion curettage	16/healed	18
I	M/1.5	CO	Hip pain, claudication and dislocation	Lesion curettage, hip reduction	12/healed	15
J	M/2.9	CO	Hip pain, claudication and subluxation	Lesion curettage, hip reduction	12/incompletely healed	17
K	M/3.4	CO	Claudication	Open biopsy	18/healed	18
L	M/13.9	BC	Hip pain, claudication and tenderness	Lesion curettage, bone allograft	36/healed	18
M	M/10.5	BC	Hip pain	Lesion curettage, bone allograft	12/healed	17
N	M/17.3	TB	Hip pain and tenderness	Lesion resection, isoniazid packing, bone allograft	24/healed	18

*BC* bone cyst, *TB* tuberculosis osteomyelitis, *EG* eosinophilic granuloma, *CO* chronic osteomyelitis, *M* male, *F* female

A summary of the imaging findings for the 14 patients is shown in Table 2. On X-ray examination, there were 3 extra-large lesions (2 cases of BC and 1 case of EG); 3 large lesions (all cases of EG); 4 medium lesions (2 cases of EG, one case of TB and one case of CO); and 4 small lesions (all cases of CO). In all, 7 children had a complete or partial sclerotic margin around the lesion (5 cases of CO, 1 case of TB, and 1 case of EG), and 7 patients showed internal and external cortical bone destruction (6 cases of EG and 1 case of CO). There were 4 cases of a periosteal reaction (all cases of EG). There were 5 cases of joint cavity involvement (4 cases of CO and 1 case of TB). There were 4 cases of joint space narrowing (all cases of CO). There were 10 cases of different degrees of osteoporosis in the femoral head. Changes in the femoral head occurred in 3 children with CO, including 1 with femoral head destruction, 1 with significant hyperplasia of the femoral head, and 1 with a smaller femoral head. Hip subluxation was found in 2 cases (both cases of CO). In 11 cases, CT examination more clearly showed the location and extent of the lesion, inner and outer cortical damage and periosteal reaction. MRI was performed in 8 children (4 cases of EG, 3 cases of CO, and 1 case of TB). MRI was helpful for observing the soft tissues surrounding the lesion. Among them, edema of the surrounding soft tissues was observed in 4 cases of EG, in only 1 of the 3 cases of CO, and not in the only case of TB.

**Table 2:** Imaging characteristics of ARL patients (following the Results)

Patient	Lesion type	X-ray								CT Cortical destruction	MRI Surrounding soft tissue edema
		size	Sclerotic margin	Boundary	Cortical destruction	Periosteal reaction	Hip joint involvement	Femoral head	Hip joint subluxation		
A	EG	L	YES	UC	YES	NO	NO	MO	NO	YES	YES
B	EG	M	NO	UC	YES	YES	NO	NO	NO	YES	–
C	EG	L	NO	UC	YES	YES	NO	O	NO	YES	YES
D	EG	M	NO	UC	YES	NO	NO	NO	NO	–	YES
E	EG	L	Partial	UC	YES	YES	NO	MO	NO	YES	–
F	EG	XL	NO	UC	YES	YES	NO	O	NO	YES	YES
G	CO	M	Partial	UC	NO	NO	NO	NO	NO	YES	–
H	CO	S	YES	C	NO	NO	YES	O	NO	NO	–
I	CO	S	YES	C	YES	NO	YES	Smaller, O	YES	YES	NO
J	CO	S	YES	C	NO	NO	NO	Larger, O	YES	–	YES
K	CO	S	YES	C	NO	NO	YES	Destruction, O	NO	NO	NO
L	BC	XL	NO	C	NO	NO	NO	NO	NO	–	–
M	BC	XL	NO	C	NO	NO	NO	MO	NO	NO	–
N	TB	M	YES	C	NO	NO	YES	MO	NO	NO	NO

*MO* mild osteoporosis, *O* osteoporosis, *BC* bone cyst, *TB* tuberculosis osteomyelitis, *EG* eosinophilic granuloma, *CO* chronic osteomyelitis, *S* small, *M* medium, *L* large, *XL* extra large, *UC* unclear, *C* clear

### Treatment and follow-up

In all 14 children, the diagnosis was confirmed by pathology; there were 6 cases of EG, 5 cases of CO, 2 cases of BC, and 1 case of TB. The 5 patients with EG underwent lesion curettage and bone grafting, and the other EG patient with multiple lesions underwent open biopsy followed by chemotherapy for 6 months (prednisolone and vindesine). Three patients with CO underwent lesion curettage, 2 of whom with hip dislocation underwent hip reduction; 2 patients with CO only underwent biopsy, incisional biopsy in 1 case and needle biopsy in the other case. Two children with BC underwent lesion curettage and allogeneic bone grafting. One patient with TB underwent lesion curettage and isoniazid implantation followed by anti-TB treatment. Except for the two patients who underwent open and needle biopsy, other patients underwent frozen biopsy during surgery.

At the 1-year follow-up, 13 patients showed complete resolution of pain and claudication, and none of them showed symptom recurrence, with an MHHS of more than 90 points and a d’Aubigné-Postel score of 17 points. Only one CO patient with pathological subluxation still had mild claudication at the last follow-up, with an MHHS of 85 points and a d’Aubigné-Postel score of 15 points. On X-ray examination, 1 patient with CO and 2 patients with BC still showed partial lesions at the last follow-up. The other 11 patients showed complete lesion healing and reconstruction.

We selected 4 cases of lesions of different pathological types as representatives to describe their diagnosis and treatment and demonstrate in more detail the clinical and imaging manifestations, treatment methods and prognosis of children with various pathological ARLs.

### Case 1 (patient C, EG)

**Fig. 1:**
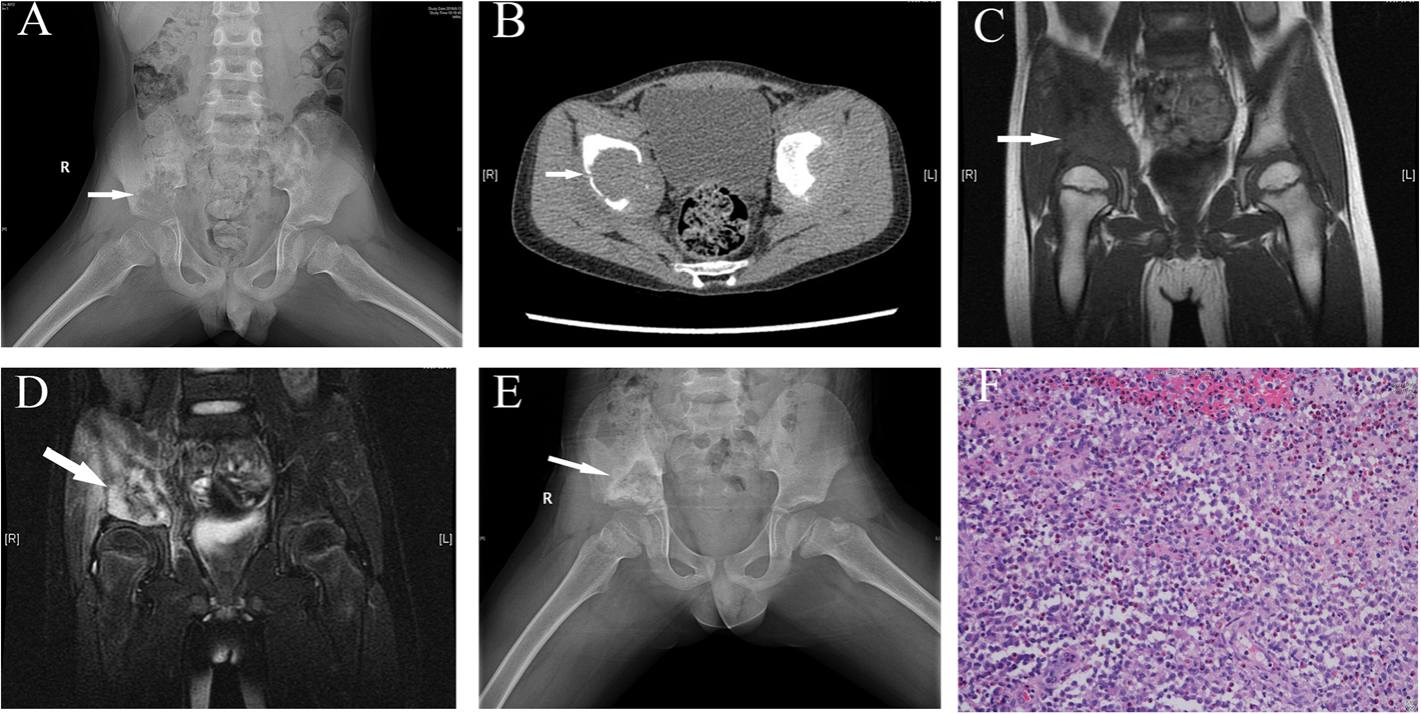
**a-f** A 4.8-year-old male patient (case C). The X-ray and CT results show right iliac bone destruction, with a low-density shadow, a blurred edge and a small periosteal reaction. Surrounding muscle tissue is swollen (**a, b**). MRI shows equal long T1 and abnormal T2 signals with cortical damage, surrounding soft tissue thickening and swelling, with long T1 and T2 signals (**c, d**). Six months after the operation, the X-ray results show partial bone growth in the lesion (**e**). The histological results suggest eosinophilic infiltration (**f**) (white arrows indicate the site of the lesion)

A 4.8-year-old boy presented with pain and activity limitation in the right hip lasting for 10 days. On physical examination, he was found to have hip tenderness and limited hip movement with an MHHS score of 75. His WBC count was 10.1*10^9/L. Radiography showed destruction of the right iliac bone, with a low-density shadow, a blurred boundary and a small periosteal reaction. CT showed cortical destruction. MRI showed equal strong T1 and abnormal T2 signals with cortical damage, surrounding soft tissue thickening and swelling, with long T1 and T2 signals (Fig. 1a-f). The child was treated with lesion curettage and allogeneic bone grafting, followed by bed rest and immobilization postoperatively. His histopathological diagnosis was EG. The patient did not bear weight on the pelvis for 1 month. Six months later, he showed complete functional recovery of the hip and no pain. X-ray examination showed partial healing of the lesion. The MHHS was greater than 95 points, and the d’Aubigné-Postel score was greater than 17 points.

### Case 2 (patient J, CO)

**Fig. 2:**
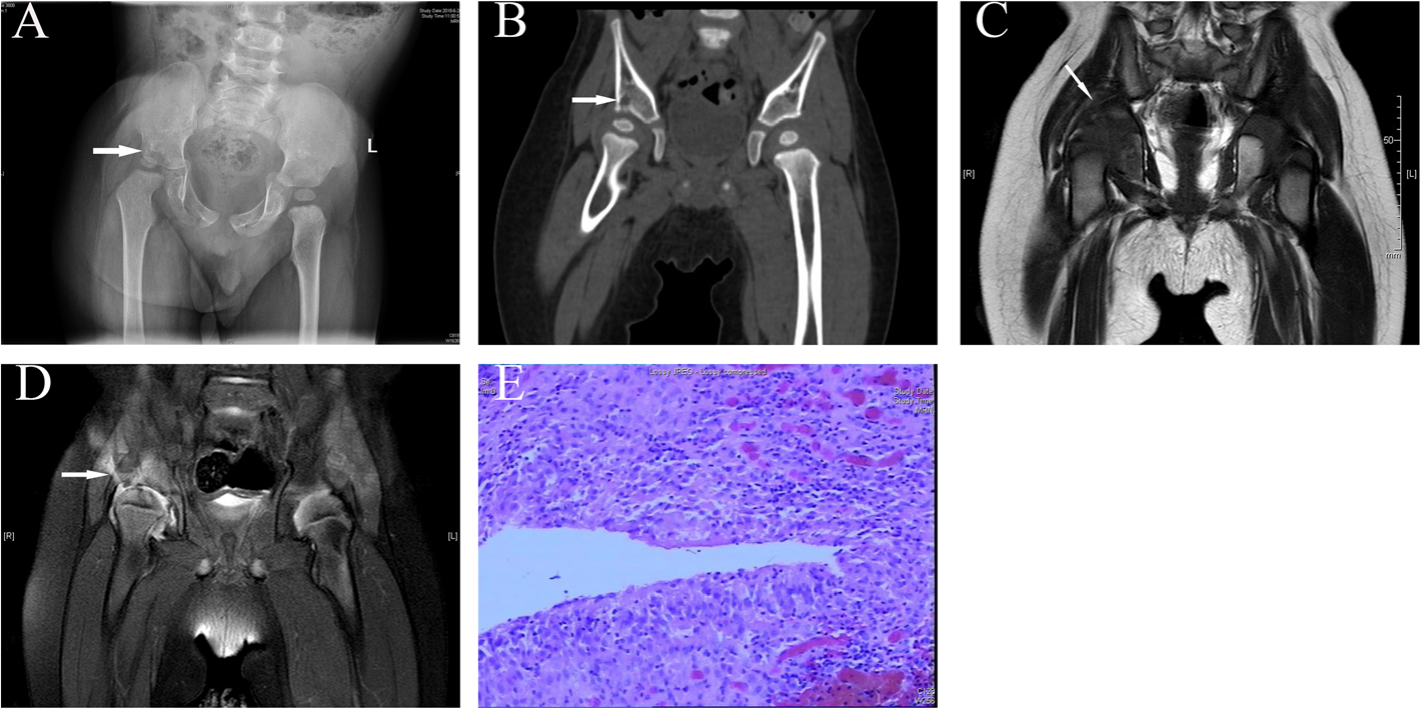
**a-e** A 2.9-year-old male patient (case J). The X-ray and CT results show right acetabular bone destruction, with a low-density shadow and hip subluxation (**a, b**). MRI shows patchy, slightly long T1 and slightly long T2 signals in the right ischial and iliac bone and long T2 signals in the right hip joint space (**c, d**). The pathological results suggest chronic inflammatory cell infiltration and granulomatous inflammation with a multinucleated giant cell response (**e**) (white arrows indicate the site of the lesion)

A 2.9-year-old boy presented with chronic right hip pain lasting for 1 year. His ESR was 17 mm/h; CRP, less than 8 mg/L; PCT, 0.342 ng/L; and WBC count, 14.7*10^9/L. Physical examination revealed lower limbs of unequal length (pelvic tilt) and restricted right hip movement. X-ray examination and CT revealed a destructive lesion in the right acetabular roof with hip subluxation. MRI showed patchy, slightly long T1 signals and slightly long T2 signals in the right ischial and iliac bone, as well as long T2 signals in the right hip joint space (Fig. 2a-e). He underwent open biopsy and lesion curettage. Pathological examination suggested chronic inflammatory cell infiltration and granulomatous inflammation with a multinucleated giant cell response. Clinical relief was achieved after the operation. However, the patient still had intermittent hip pain and a slight limp after the operation. One year after the operation, the MHHS was 85 points, and the d’Aubigné-Postel score was 15 points.

### Case 3 (patient M, BC)

**Fig. 3:**
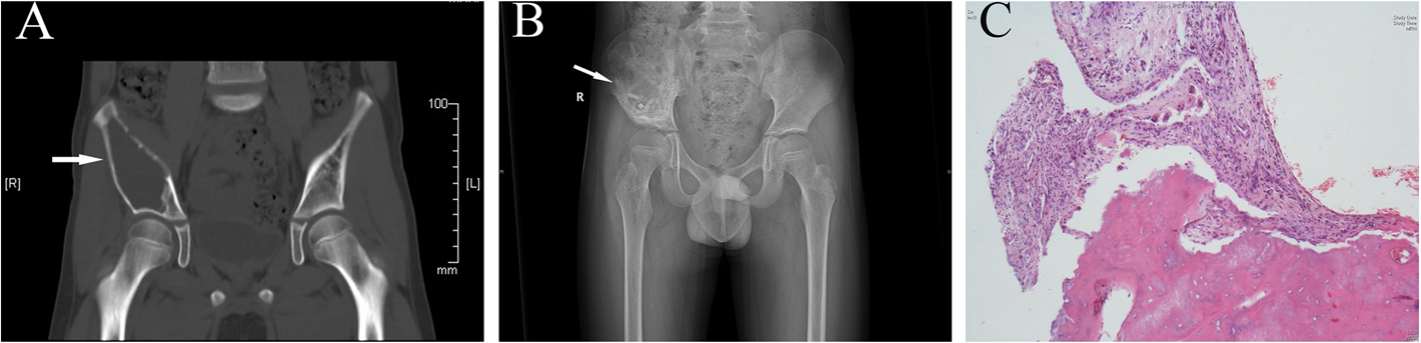
**a-c** A 10.5-year-old male patient (case M). The CT results show right iliac bone destruction, large, oval, low-density shadows, cortical thinning, and partial discontinuity of the iliac cortical bone (**a**). The X-ray (**b**) results show reconstruction of the ilium after 1 year. The histological appearance of the lesion shows aneurysmal BCs (**c**) (white arrows indicate the site of the lesion)

A 10.5-year-old boy presented with right hip pain lasting for 5 months, and physical examination only showed tenderness in the right hip. X-ray examination and CT showed a single-cystic, expansive lesion occupying almost the whole right ilium, with a clear boundary and thin and discontinuous cortical bone (Fig. 3a-c). He was diagnosed with BC before surgery. He underwent lesion curettage and bone grafting. During the operation, the capsule cavity was observed to be filled with pale yellow liquid. One year after the operation, partial healing of the lesion was observed on X-ray examination. The MHHS was greater than 90 points, and the d’Aubigné-Postel score was greater than 17 points.

### Case 4 (patient N, TB)

**Fig. 4:**
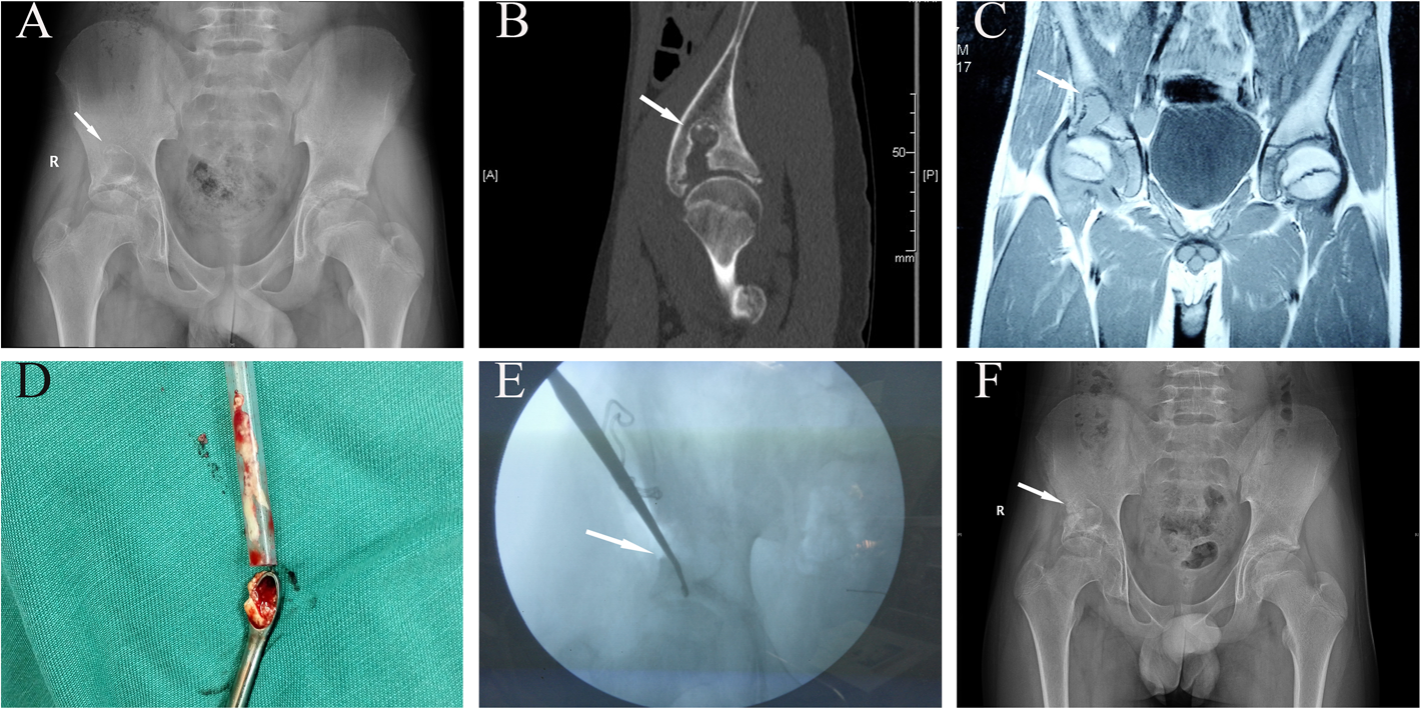
**a-f** A 17.3-year-old male patient (case N). The X-ray and CT results show right iliac bone destruction, with partial absence of the acetabulum, a clear boundary, and a sclerotic margin (**a, b**). MRI shows slightly long T1 signals in the right acetabular roof (**c**). During the operation, the lesion was found to consist of caseous tissue (**d**), and the X-ray results show that the lesion was cleared (**e**). The X-ray (**f**) results show partial bone growth after 6 months (white arrows indicate the site of the lesion)

A 17.3-year-old boy presented with right hip pain lasting for 3 years, and his physical examination was abnormal; the MHHS was 82. On admission, his ESR was 55 mm/h; CRP, less than 8 mg/L; and WBC count, 11.2*10^9/L. The interferon-gamma release assay was positive. X-ray examination showed a patchy, low-density lesion, with a clear and sclerotic boundary and a blurry hip joint space. CT showed damage to the right acetabulum and part of the internal femoral head. MRI showed long, abnormal T1 signals in the right ilium and hip joint cavity (Fig. 4a-f). He was diagnosed with TB osteomyelitis. The patient underwent lesion curettage and bone grafting. During the operation, the lesion was observed to be filled with caseous tissue. The lesion was removed, isoniazid was placed in the lesion site, and allogenic bone was grafted. Regular anti-TB treatment was performed after the operation. X-ray examination indicated complete healing of the lesion 2 years after the operation. At the latest fallow-up, the MHHS was 90 points, and the d’Aubigné-Postel score was 18 points.

## Discussion

**Fig. 5:**
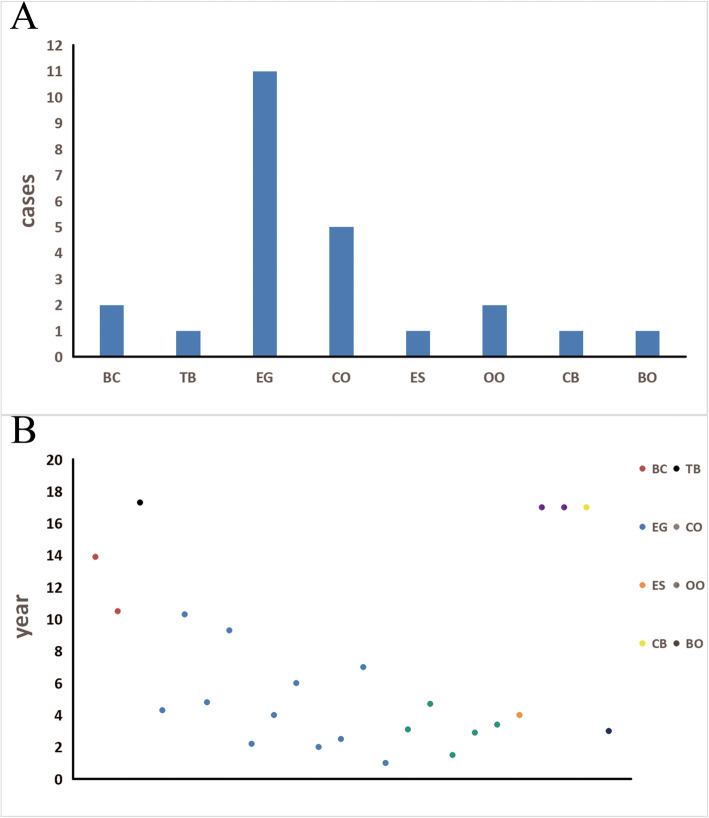
**a-b** These graphs show the distribution of ARLs according to (**a**) diagnosis and (**b**) age. BC, bone cyst; TB, tuberculosis osteomyelitis; EG, eosinophilic granuloma; CO, chronic osteomyelitis; ES, Ewing’s sarcoma; OO, osteoid osteoma; CB, chondroblastoma; BO, Bartonella osteomyelitis

The acetabular roof is formed by expansion of the lower end of the ilium, accounting for 2/5 of the acetabulum. It is the main weight-bearing part of the hip joint in supporting the weight of the trunk. Fractures or tumors affecting the acetabular roof are of special concern, and the treatment of such lesions is difficult and complex. ARLs are rare and have mostly been described in case reports. We used ‘acetabular roof’ and ‘child’ as the key words to screen all English articles on ARLs. A total of 8 articles were found; of them, 6 were case reports, with a total of 10 cases. There were 5 cases of EG, 2 cases of osteoid osteoma (OO), 1 case of ES, 1 case of chondroblastoma (CB), and 1 case of Bartonella osteomyelitis (BO) (Table 3). Combined with the cases presented in this article, we collected a total of 24 cases of ARL, the distribution of which is shown in Fig. 5a. According to the available data, most of the ARLs were benign, and there was only one case of ES. EG was the most common lesion (45%), followed by chronic inflammation (20%). The age distribution is shown in Fig. 5b. The age distribution of children with EG was wider, ranging from 1 to 10 years old and mainly centered around the ages of 1 to 5 years. Chronic inflammatory lesions were most common in children less than 5 years old, which may be caused by a missed diagnosis in the acute stage due to the unclear expression of painful areas in younger children, the deep location of the lesion, concealment by clothing, and subtle signs compared with those of pyogenic hip arthritis. Additionally, the pathogenic bacteria of acetabular osteomyelitis are mostly of low toxicity [15] and can be controlled by conventional antibiotics used for signs of infection considered to indicate general infection; thus, an insufficient treatment duration leads to chronic disease progression [16].

**Table 3:** Pediatric cases of ARL found on literature review (following the Discussion)

Reference	Year	Patients; age	Sex; side	Lesion location	Symptom	Treatment	Follow-up	Results	Lesion type
Zoccali et al. [9]	2017	1; 17 y	M; R	Triradiate cartilage	Right hip with onset occurring over approximately 2 months	Intralesional excision consisting of curettage with local phenol adjuvant treatment	4 y	Right hip with onset occurring over approximately 2 months	CB
Benyass et al. [10]	2016	1; 17 y	M; R	Acetabular roof	Pain in right hipwith lameness	Complete percutaneous resection of the nidus under imaging guidance	1 y	Complete healing with total and definitive disappearance of symptoms after 1 year	OO
Puri et al. [11]	2015	1; 3 y	F; R	Acetabulum	History (5 days) of pain in right leg and refusal to bear weight	Antibiotics (rifampinand azithromycin)	2 m	History (5 days) of pain in right legand refusal to bear weight	BO
Bosschaert et al. [12]	2010	1; 17 y	F; L	Weight-bearing surface of the acetabulum	Chronic pain in left hip, with nocturnal episodes	Radiofrequency ablation	1 y	Chronic pain in left hip, with nocturnal episodes	OO
Ando et al. [2]	2008	2; 4 and 6 y	2 M;1 R/1 L	Acetabulum or ilium	Pain in right thigh and limping	Needle biopsy or open biopsy	3 m and 1 y	Complete healing	EG
Verma et al. [13]	2004	1; 4 y	F; L	Acetabular roofto the sacroiliac joint	Left hip pain and left-sided limp	Resection followed by osteoarticular allograftreconstruction	2 y	Disease-free with an excellent functional outcome	ES
Howard et al. [3]	1996	1; 7 y	F; R	Right ilium	Pain and limp in the right lower limb	Curettage of the lesion	8 m	Healed	EG
NITTER et al. [14]	1956	2; 2 and 2.5 y	2 F; 1 R/1 L	Ilium	Pain in the abdomen and left leg with limp	Roentgen therapy or lesion removal	1 and 3 y	Complete healing	EG

*BC* bone cyst, *TB* tuberculosis osteomyelitis, *EG* eosinophilic granuloma, *CO* chronic osteomyelitis, *ES* Ewing’s sarcoma, *OO* osteoid osteoma, *CB* chondroblastoma, *BO* Bartonella osteomyelitis, *Y* year, *mo* month, *F* femur, *M* male, *L* left, *R* right

Although ARLs mainly consist of EG and CO lesions, various other types of lesions can occur, and even desmoid tumors have been reported in adults [5]. The levels of laboratory indicators of inflammation (WBC, CRP, ESR, and PCT) may be elevated in children with CO and EG, but they are of little help for differential diagnosis. High-resolution CT can confirm the diagnosis of OO [17]. MRI (uniform high T2 signals in the lesion) combined with X-ray examination can confirm the diagnosis of BC in most cases. CB mostly originates from triradiate cartilage, which is meaningful for differential diagnosis. The imaging features of EG vary according to the disease stage, and EG lesions can resemble any other kind of lesion. In the acute stage, EG is characterized by malignant manifestations: cortical destruction, a periosteal reaction, a blurred border, and surrounding soft tissue edema, but soft tissue masses are rare on physical examination and MRI, and these can be distinguished from malignant tumors. In the chronic phase, EG mainly shows benign manifestations, with a clear boundary and sclerotic margin. In the transitional phase, EG lesions may have all of the above characteristics. In the current literature, we found that BC (including aneurysmal BC and simple BC) and ES lesions were mostly very large [18, 19] and that most of these lesions involved areas beyond the acetabular roof. EG lesions were mostly large, although oversized EG lesions occurred in some cases. In our series, the child with an oversized EG lesion happened to have multiple lesions. CO and TB osteomyelitis manifest as small and medium lesions with a clear boundary. Joint space narrowing, connection of the lesion with the joint cavity, and femoral head involvement or hypertrophy are more supportive of CO. When TB osteomyelitis is suspected, a history of TB or exposure and an interferon-gamma release assay can help to confirm the diagnosis before surgery [20, 21].

The treatment of ARLs varies due to the diversity of the lesions. If OO can be diagnosed definitively, CT-guided radiofrequency ablation is preferred for minimally invasive treatment, but attention should be paid to protection of the articular cartilage [17]. For BC lesions that can be diagnosed by MRI, puncture extraction can be helpful to differentiate simple BC from aneurysmal BC. Injection treatment is performed for small lesions, and lesion curettage and bone grafting are proposed for large lesions that are prone to pathological fracture [22]. If the diagnosis cannot be confirmed by laboratory and imaging examinations, puncture biopsy should be performed when a malignant lesion is suspected. If a malignant tumor is diagnosed, surgical resection and chemotherapy should be performed according to the corresponding therapeutic principle. If a malignant lesion can be excluded, open biopsy, curettage, and bone grafting are recommended for larger lesions. Although there have been some reported cases of EG lesion self-healing after open or needle biopsy [23, 24], this process takes a long time, during which children need to limit their movement to avoid pathological fracture. The treatment of chronic pelvic osteomyelitis is mainly based on surgical methods, and simple antibiotic treatment is often ineffective [25]. If there is no obvious accumulation of pus and no pathogenic bacteria can be cultured, it is controversial whether antibiotics should be applied after lesion curettage. Three patients in this group recovered well without antibiotic treatment after the operation. There was still one child with severe symptoms preoperatively whose hip function was poor at the last follow-up even after antibiotic treatment was performed. The key to the treatment of acetabular roof CO is wide surgical debridement and prolonged bed rest [26]. In cases with cultured pathogenic bacteria, sufficient treatment with effective antibiotics is necessary. Compared with ARLs in adults, ARLs in children are less likely to be malignant tumors, and most are benign lesions. Bauones et al. [6] reported 3 cases of malignant acetabular roof tumor bone metastasis in adults. The most common surgical method applied was curettage, and radical resection was not required. If there is a high degree of suspicion of malignancy or frozen biopsy is performed during surgery, an oncologist can be invited to diagnose and treat the lesion. Adult ARLs are mostly malignant tumors, and these tumors may be the primary lesion, but most of them are metastases from other malignant tumors. Radical resection of the lesion after chemotherapy is often performed, and the prognosis is generally poor. A small number of adults have benign tumors, such as desmoid tumors and OO lesions, and recover well after surgery.

## Conclusion

ARLs in children are uncommon. In this series of 14 children, EG and nonspecific CO were the most common forms of ARL. These lesions had no characteristic imaging findings. Although all of our cases had a benign etiology, one must always suspect and exclude malignancy. The diagnosis is mainly based on histopathological examination. Surgical treatment can be effective, with good clinical outcomes at 12 months.

## Data Availability

The datasets used and analysed during the current study are available from the corresponding author on reasonable request.

## Abbreviations

ARL: Acetabular roof lesion
EG: Eosinophilic granuloma
CT: Computed tomography
MRI: Magnetic resonance imaging
CO: Chronic osteomyelitis
BC: Bone cyst
TB: Tuberculosis
PCT: Procalcitonin high-sensitivity
CRP: C-reactive protein
ESR: Erythrocyte sedimentation rate
MHHS: Modified Harris Hip Score
WBC: White blood cell
OO: Osteoid osteoma
ES: Ewing’s sarcoma
CB: Chondroblastoma
BO: Bartonella osteomyelitis
NHL: Non-Hodgkin’s lymphoma
NB: Neuroblastoma

## References

[1] Enneking WF, Dunham WK (1978). Resection and reconstruction for primary neoplasms involving the innominate bone. J Bone Joint Surg Am.

[2] Ando A, Hatori M, Hosaka M (2008). Eosinophilic granuloma arising from the pelvis in children: a report of three cases. Ups J Med Sci.

[3] Howard CB, Nyska M, Porat S (1996). Solitary eosinophilic granuloma of the pelvis in children. A report of three cases. Arch Orthop Trauma Surg.

[4] Sun X, Lou Y, Wang X (2016). The diagnosis of iliac bone destruction in children: 22 cases from two Centres. Biomed Res Int.

[5] Prasad H, Jagadesh G (2013). Desmoid tumor of Ilio-Acetabular region with articular cartilage breach: a case report. J Orthop Case Rep.

[6] Bauones S, Garnon J, Chari B (2018). Protection of the proximal articular cartilage during percutaneous thermal ablation of Acetabular metastasis using temperature monitoring. Cardiovasc Intervent Radiol.

[7] Welker JA, Henshaw RM, Jelinek J (2000). The percutaneous needle biopsy is safe and recommended in the diagnosis of musculoskeletal masses. Cancer..

[8] D'Aubigne RM, Postel M (1954). Functional results of hip arthroplasty with acrylic prosthesis. J Bone Joint Surg Am.

[9] Zoccali C, Arrigoni F, Mariani S (2017). An unusual localization of chondroblastoma: the triradiate cartilage; from a case report a reconstructive technique proposal with imaging evolution. J Clin Orthop Trauma.

[10] Benyass Y, Chafry B, Koufagued K (2016). Osteoid osteoma of the acetabular roof: a case report. J Med Case Rep.

[11] Puri K, Kreppel AJ, Schlaudecker EP (2015). Bartonella osteomyelitis of the acetabulum: case report and review of the literature. Vector Borne Zoonotic Dis.

[12] Bosschaert PP, Deprez FC (2010). Acetabular osteoid osteoma treated by percutaneous radiofrequency ablation: delayed articular cartilage damage. JBR-BTR..

[13] Verma NN, Kuo KN, Gitelis S (2004). Acetabular osteoarticular allograft after Ewing's sarcoma resection. Clin Orthop Relat Res.

[14] Nitter L (1956). Three cases of eosinophilic granuloma of the pelvis in children. Acta Radiol.

[15] Ramaesh R, Gaston MS, Simpson AH (2013). Chronic osteomyelitis of the pelvis. Acta Orthop Belg.

[16] Jain M, Sarkar S, Naik S (2018). Iliac bone tuberculosis with bicompartmental abscess. BMJ Case Rep.

[17] Rosenthal DI, Hornicek FJ, Torriani M (2003). Osteoid osteoma: percutaneous treatment with radiofrequency energy. Radiology..

[18] Sharifah M, Nurhazla H, Suraya A (2011). Pelvic aneurysmal bone cyst. Biomed Imaging Interv J.

[19] Cottalorda J, Chotel F, Kohler R (2005). Aneurysmal bone cysts of the pelvis in children: a multicenter study and literature review. J Pediatr Orthop.

[20] Watts HG, Lifeso RM (1996). Tuberculosis of bones and joints. J Bone Joint Surg Am.

[21] Sester M, Sotgiu G, Lange C (2011). Interferon-γ release assays for the diagnosis of active tuberculosis: a systematic review and meta-analysis [published correction appears in Eur Respir J. 2012 mar;39(3):793]. Eur Respir J.

[22] Yildirim E, Ersözlü S, Kirbaş I (2007). Treatment of pelvic aneurysmal bone cysts in two children: selective arterial embolization as an adjunct to curettage and bone grafting. Diagn Interv Radiol.

[23] Arkader A, Glotzbecker M, Hosalkar HS (2009). Primary musculoskeletal Langerhans cell histiocytosis in children: an analysis for a 3-decade period. J Pediatr Orthop.

[24] Corby RR, Stacy GS, Peabody TD (2008). Radiofrequency ablation of solitary eosinophilic granuloma of bone. AJR Am J Roentgenol.

[25] Maraqa NF, Gomez MM, Rathore MH (2002). Outpatient parenteral antimicrobial therapy in osteoarticular infections in children. J Pediatr Orthop.

[26] Beslikas TA, Panagopoulos PK, Gigis I (2005). Chronic osteomyelitis of the pelvis in children and adolescents. Acta Orthop Belg.

